# Improved water, sanitation, hygiene and waste services in health-care facilities, Ukraine

**DOI:** 10.2471/BLT.24.291716

**Published:** 2024-12-03

**Authors:** Maggie Montgomery, Arkadii Vodianyk, Nataliia Piven, Arabella Hayter, Ryan Schweitzer, Tetyana Skapa, Bruce Gordon, Oliver Schmoll

**Affiliations:** a Water, Sanitation, Hygiene and Health Unit, World Health Organization, Avenue Appia 20, 1211 Geneva 27, Switzerland.; b Country Office, World Health Organization, Kyiv, Ukraine.; c Centers for Disease Control and Prevention, Atlanta, United States of America.; d Ministry of Health, Kyiv, Ukraine.; e Water and Climate, World Health Organization European Centre for Environment and Health, Bonn, Germany.

## Abstract

**Problem:**

Water, sanitation and waste infrastructure and services in Ukrainian health-care facilities often fail to meet global and national standards, hindering the provision of safe, quality care. The war has worsened existing problems.

**Approach:**

To incrementally improve water, sanitation, hand hygiene, environmental cleaning and health-care waste practices, the World Health Organization (WHO) is working with the health ministry, the Ukrainian Public Health Centre and regional United States Centers for Disease Prevention and Control (CDC) to implement the Water and Sanitation for Health Facility Improvement Tool (WASH FIT). In December 2022, WHO trained staff from the regional CDC, Ukrainian Public Health Centre and nine priority health-care facilities. Facility teams assessed services, and developed and implemented improvement plans in January 2023. Follow-up assessments were conducted after six months.

**Local setting:**

About 4300 health-care facilities operate in Ukraine. Funding for new water and sanitation infrastructure, as well as operating and maintaining existing services, is likely inadequate.

**Relevant changes:**

After implementation, the average WASH FIT score increased from 66 out of a maximum of 100 (range: 57–73) to 83 (range: 69–93). Facilities more effectively managed water-related risks, replaced broken taps, improved waste management practices and installed backup water supply and storage sources.

**Lessons learnt:**

WASH FIT can be used to identify gaps in water, sanitation, hygiene and waste services and track progress towards addressing these gaps. Engaged local leaders, health-based national water, sanitation, hygiene standards, regular on-site training and mentoring drive these improvements. Strengthening public health entities is critical to institutionalize the process.

## Résumé

**Problème:**

Les infrastructures et les services d’approvisionnement en eau, d’assainissement et de gestion des déchets dans les établissements de soins de santé ukrainiens ne satisfont souvent pas aux normes mondiales et nationales en la matière, ce qui entrave la fourniture de soins sûrs et de qualité. De plus, la guerre a aggravé les problèmes existants.

**Approche:**

Afin d’améliorer progressivement les pratiques en matière d’approvisionnement en eau, d’assainissement, d’hygiène des mains, de nettoyage de l’environnement et de déchets d’activités de soins, l’Organisation mondiale de la santé (OMS) coopère avec le ministère de la Santé ukrainien, le Centre ukrainien de santé publique et les Centres régionaux de prévention et de contrôle des maladies (CDC) pour mettre en œuvre l’Outil d’amélioration de l’eau, de l’assainissement et de l’hygiène dans les établissements de santé (WASH FIT). En décembre 2022, l’OMS a formé le personnel des CDC régionaux, du Centre ukrainien de santé publique et de neuf établissements de soins de santé prioritaires. Les équipes de ces établissements ont évalué les services et élaboré et mis en œuvre des plans d’amélioration en janvier 2023. Des évaluations de suivi ont été réalisées au bout de six mois.

**Environnement local:**

L’Ukraine compte environ 4 300 établissements de soins de santé. Le financement des nouvelles infrastructures d’approvisionnement en eau et d’assainissement ainsi que du fonctionnement et de l’entretien des services existants est probablement insuffisant.

**Changements significatifs:**

Après la mise en œuvre, le score WASH FIT moyen est passé de 66 sur un maximum de 100 (fourchette: 57–73) à 83 (fourchette: 69–93). Les établissements ont géré plus efficacement les risques liés à l’eau, remplacé les robinets cassés, amélioré les pratiques de gestion des déchets et installé des sources d’approvisionnement en eau et de stockage de secours.

**Leçons tirées:**

WASH FIT permet d’identifier les lacunes dans les services d’approvisionnement en eau, d’assainissement, d’hygiène et de gestion des déchets et de faire le suivi des progrès réalisés dans la résolution de ces lacunes. Des dirigeants locaux engagés, des normes nationales en matière d’eau, d’assainissement et d’hygiène fondées sur la santé, des formations régulières dispensées sur place ainsi qu’un encadrement sont à l’origine de ces améliorations. Le renforcement des agences de santé publique est essentiel pour institutionnaliser ce processus.

## Resumen

**Situación:**

Las infraestructuras y servicios de agua, saneamiento y residuos de los centros sanitarios ucranianos no suelen cumplir los estándares mundiales y nacionales, lo que dificulta la prestación de una atención segura y de calidad. Además, la guerra ha agravado los problemas existentes.

**Enfoque:**

Para mejorar progresivamente las prácticas de agua, saneamiento, higiene de las manos, limpieza ambiental y residuos sanitarios, la Organización Mundial de la Salud (OMS) colabora con el Ministerio de Sanidad, el Centro de Salud Pública de Ucrania y los Centros para la Prevención y el Control de Enfermedades (CDC) regionales en la aplicación de la Herramienta de agua y saneamiento para la mejora de los centros sanitarios (WASH FIT). En diciembre de 2022, la OMS formó a personal de los CDC regionales, del Centro de Salud Pública de Ucrania y de nueve centros sanitarios prioritarios. Los equipos del centro evaluaron los servicios y elaboraron e implementaron planes de mejora en enero de 2023. Se realizaron evaluaciones de seguimiento al cabo de seis meses.

**Marco regional:**

Cerca de 4300 centros sanitarios funcionan en Ucrania. Es probable que la financiación de nuevas infraestructuras de agua y saneamiento, así como el funcionamiento y mantenimiento de los servicios existentes, sea insuficiente.

**Cambios importantes:**

Tras la implementación, la puntuación media de WASH FIT aumentó de 66 sobre un máximo de 100 (rango: 57-73) a 83 (rango: 69-93). Los centros gestionaron más eficientemente los riesgos relacionados con el agua, sustituyeron los grifos averiados, mejoraron las prácticas de gestión de residuos e instalaron fuentes de suministro y almacenamiento de agua de reserva.

**Lecciones aprendidas:**

WASH FIT puede utilizarse para identificar carencias en los servicios de agua, saneamiento, higiene y gestión de residuos, así como para realizar un seguimiento de los avances en la resolución de estas carencias. Los líderes locales comprometidos, los estándares nacionales de agua, saneamiento e higiene basados en la salud, la formación periódica en el lugar y la tutoría impulsan estas mejoras. El fortalecimiento de las entidades de salud pública es fundamental para institucionalizar el proceso.

## ملخص

**المشكلة:**

إن البنية التحتية والخدمات الخاصة بالمياه، والصرف الصحي، والنفايات في مرافق الرعاية الصحية الأوكرانية، غالبًا ما تفشل في الوفاء بالمعايير العالمية والوطنية، مما يعيق تقديم رعاية آمنة وجيدة الجودة. وقد أدت الحرب إلى تفاقم المشاكل القائمة.

**الأسلوب:**

لتحسين ممارسات المياه، والصرف الصحي، ونظافة اليدين، والتنظيف البيئي، والنفايات الصحية بشكل تدريجي، تعمل منظمة الصحة العالمية (WHO)، مع وزارة الصحة، ومركز الصحة العامة الأوكراني، والمراكز الإقليمية للوقاية من الأمراض والسيطرة عليها (CDC)، لتنفيذ أداة المياه والصرف الصحي لتحسين المرافق الصحية (WASH FIT). في ديسمبر/كانون أول 2022، قامت منظمة الصحة العالمية بتدريب فريق العمل في المراكز الإقليمية للوقاية من الأمراض والسيطرة عليها (CDC)، ومركز الصحة العامة الأوكراني، وتسعة من مرافق الرعاية الصحية ذات الأولوية. قامت فرق المرافق بتقييم الخدمات، وتطوير وتنفيذ خطط التحسين في يناير/كانون ثاني 2023. وأُجريت تقييمات المتابعة بعد ستة أشهر.

**المواقع المحلية:**

يوجد حوالي 4300 مرفق رعاية صحية في أوكرانيا قيد التشغيل. من المرجح أن يكون التمويل اللازم للبنية التحتية الجديدة للمياه والصرف الصحي، غير كاف، فضلاً عن تشغيل وصيانة الخدمات القائمة.

**التغيّرات ذات الصلة:**

بعد التنفيذ، ارتفعت درجة WASH FIT المتوسطة من 66 من أصل 100 كحد أقصى (المدى: 57 إلى 73) إلى 83 (المدى: 69 إلى 93). تمكنت المرافق من إدارة المخاطر المتعلقة بالمياه بشكل أكثر فعالية، واستبدال الصنابير المكسورة، وتحسين ممارسات إدارة النفايات، وتركيب مصادر إمدادات المياه الاحتياطية والتخزين.

**الدروس المستفادة:**

يمكن استخدام WASH FIT لتحديد الثغرات في خدمات المياه، والصرف الصحي، والنظافة، والنفايات، وتتبع التقدم نحو معالجة هذه الثغرات. إن القادة المحليين المشاركين، والمعايير الوطنية القائمة على الصحة للمياه، والصرف الصحي، والنظافة، والتدريب الميداني المنتظم، والتوجيه، يدفعون في اتجاه هذه التحسينات. إن دعم كيانات الصحة العامة أمر بالغ الأهمية لإضفاء الطابع المؤسسي على العملية.

## 摘要

**问题:**

乌克兰卫生保健机构中的供水、环境卫生和废物管理基础设施和服务往往无法达到全球和国家标准，这阻碍了其提供安全、优质的医疗服务。战争加剧了现有的这些问题。

**方法:**

为了逐步改善供水、环境卫生、手卫生、环境清洁和卫生保健废物管理实践，世界卫生组织 (WHO) 正在与卫生部、乌克兰公共卫生中心和区域疾病预防控制中心 (CDC) 合作，推行卫生机构中水卫生服务改进工具 (WASH FIT)。2022 年 12 月，WHO 对来自区域 CDC、乌克兰公共卫生中心和 9 个重点卫生保健机构的工作人员进行了培训。由卫生机构工作人员组成的团队于 2023 年 1 月对相关服务进行了评估，并制定和推行了改进计划。六个月后进行了追踪评估。

**当地状况:**

乌克兰约有 4,300 家卫生保健机构。用于新建供水和环境卫生基础设施以及运营和维护现有服务的资金可能不足。

**相关变化:**

改进计划推行后，WASH FIT 平均得分从 66 分（满分 100 分，范围：57-73 分）提高到了 83 分（范围：69-93 分）。卫生机构可以更有效地管理与水相关的风险、更换损坏的水龙头、改进废物管理实践，以及安装备用供水和储水设备。

**经验教训:**

WASH FIT 可用于确定在供水、环境卫生、个人卫生和废物管理服务方面存在的差距，并跟踪解决这些差距的进展情况。在当地领导人的积极参与下，通过制定基于健康的国家供水、环境卫生、个人卫生标准，并定期提供现场培训和指导，有力推动了改进工作进程。加强公共卫生实体建设对于将这一过程制度化至关重要。

## Резюме

**Проблема:**

Инфраструктура и услуги в сфере водоснабжения, санитарии и утилизации отходов в медицинских учреждениях Украины часто не соответствуют мировым и национальным стандартам, что препятствует безопасному и качественному оказанию медицинской помощи. В ходе войны существующие проблемы усугубились.

**Подход:**

Для постепенного повышения качества водоснабжения, водоотведения, гигиены рук, уборки окружающей среды и утилизации медицинских отходов Всемирная организация здравоохранения (ВОЗ) совместно с Министерством здравоохранения, Украинским центром общественного здоровья и региональными Центрами профилактики и контроля заболеваний (ЦКЗ) внедрила инструмент по улучшению водоснабжения и водоотведения в медицинских учреждениях (WASH FIT). В декабре 2022 года ВОЗ провела обучение сотрудников регионального ЦКЗ, Украинского центра общественного здоровья и девяти приоритетных медицинских учреждений. В январе 2023 года коллективы учреждения провели оценку услуг, разработали и внедрили планы по улучшению. Последующие оценки были проведены через шесть месяцев.

**Местные условия:**

На Украине работает около 4300 медицинских учреждений. Вероятно, финансирование новой инфраструктуры водоснабжения и водоотведения, а также эксплуатации и обслуживания существующих услуг является недостаточным.

**Осуществленные перемены:**

После внедрения инструмента WASH FIT средний показатель вырос с 66 из 100 (диапазон: 57–73) до 83 (диапазон: 69–93). Учреждения стали эффективнее управлять рисками, связанными с водоснабжением, заменили сломанные краны, усовершенствовали методы утилизации отходов и установили резервные источники водоснабжения и хранения воды.

**Выводы:**

Инструмент WASH FIT может использоваться для выявления недостатков в предоставлении услуг по водоснабжению, водоотведению, гигиене и утилизации отходов, а также для отслеживания прогресса в устранении этих недостатков. Этому способствуют вовлеченные местные руководители, национальные стандарты в сфере водоснабжения, водоотведения и гигиены, основанные на принципах здравоохранения, регулярное обучение на рабочих местах и наставничество. Укрепление структур общественного здравоохранения имеет решающее значение для институционализации этого процесса.

## Introduction

Fully functioning and safely managed water, sanitation and health-care waste management services are essential for infection prevention and control. These services ensure quality of care and support the dignity and human rights of all patients, including in emergency contexts. Globally, one in five health-care facilities lacks basic drinking-water and one in 10 facilities lacks basic sanitation services.^1^ To facilitate regular assessments, improvements and monitoring of such services, the World Health Organization (WHO) and the United Nations Children’s Fund developed the Water and Sanitation for Health Facility Improvement tool (WASH FIT).^2^

Water and sanitation services in Ukraine have faced years of underinvestment, posing major health risks to the population. The issue has been further exacerbated by the war.^3^ Addressing this issue is critical to achieving several Ukrainian national priorities, including universal primary health care, preventing antimicrobial resistance and increasing access to vaccinations. Furthermore, as part of the European Union (EU) accession process, Ukraine is required to shift towards a health-based risk management approach to managing water and sanitation services, including in health-care facilities, to meet quality of care standards.

## Local setting

In 2024, approximately 9.6 million people in Ukraine required assistance to meet their basic water, sanitation and hygiene needs.^4^ The war has caused an estimated 11.6 billion United States dollars (US$) economic loss within the water sector due to increased operating costs and decreased consumption and revenue. In Ukraine, there are an estimated 4300 health-care facilities, with the majority being primary, and the remaining are secondary, tertiary, emergency and palliative facilities. A 2024 national census on health facility functionality found that over 90% of the facilities surveyed had basic water, sanitation and hygiene services. However, this figure is likely an overestimation because the census assessed only limited aspects of water, sanitation and hygiene. For example, the census did not include water quality or reliability nor sanitation access for all users.^5^

Despite work in 2017 to increase primary health-care accessibility, alongside health financing reform,^6^ recent data suggest low and likely inadequate expenditure to fund new infrastructure, as well as operate and maintain existing infrastructure services in health-care facilities, including water and sanitation.^7^

## Approach

Since 2022, as part of broader efforts to strengthen the resilience and safety of water and sanitation services in Ukraine, WHO and the United States Centers for Disease Control and Prevention (CDC) have supported strengthening national policies and standards as well as services for water, sanitation, hygiene and waste in health-care facilities. One component of these efforts is the implementation of WASH FIT. The tool provides a framework to assess and continuously improve and monitor water, sanitation, hygiene and waste services. The tool also addresses climate resilience, sustainability and equity of water, sanitation, hygiene and waste services. WASH FIT includes 96 indicators, which are aligned with WHO and national standards in seven areas: (i) water; (ii) sanitation; (iii) hand hygiene; (iv) cleaning in treatment and consultation areas; (v) health-care waste; (vi) energy; and (vii) facility management. By aligning with the WHO Infection Prevention and Control Assessment Framework,^8^ which Ukraine adopted nationally in 2021, the tool provides an opportunity to strengthen water, sanitation and hygiene elements of the framework.

In December 2022, the health ministry selected nine pilot health-care facilities in western and central Ukraine (four specialized and five primary care), based on needs, security access and alignment with other synergistic efforts, such as infection prevention and control and increasing access to vaccines. Facility managers, nurses, and water, sanitation and hygiene experts, along with representatives from regional CDC and the Ukrainian Public Health Centre participated in a three-day in-person training facilitated by WHO and hosted by the health ministry. The focus was on application of WASH FIT, water, sanitation, hygiene and waste interventions, and basic infection prevention and control. On average, these facilities collectively serve over 12 000 patients per month.

In March 2023, external experts on water, sanitation, hygiene and infection prevention and control conducted on-site WASH FIT baseline assessments. Multidisciplinary teams, consisting of WHO, the health ministry and nongovernmental organizations, provided three two-hour online and two onsite one-day training and coaching sessions for nurses, doctors, facility managers and facility maintenance personnel on how to develop water, sanitation and hygiene improvement plans for health-care facilities. Subsequently, the multidisciplinary teams developed and implemented these plans, with support from facility managers, in health-care facilities. WHO procured equipment based on facility needs, including waste bins, cleaning and hand hygiene supplies and laundry equipment. Total investment was approximately US$ 10 000 per facility. Training and follow-up visits totalled an additional US$ 20 000. After provision of technical support and equipment, in July 2023, a second round of external assessment was conducted to assess improvements. 

## Relevant changes

**Fig. 1:**
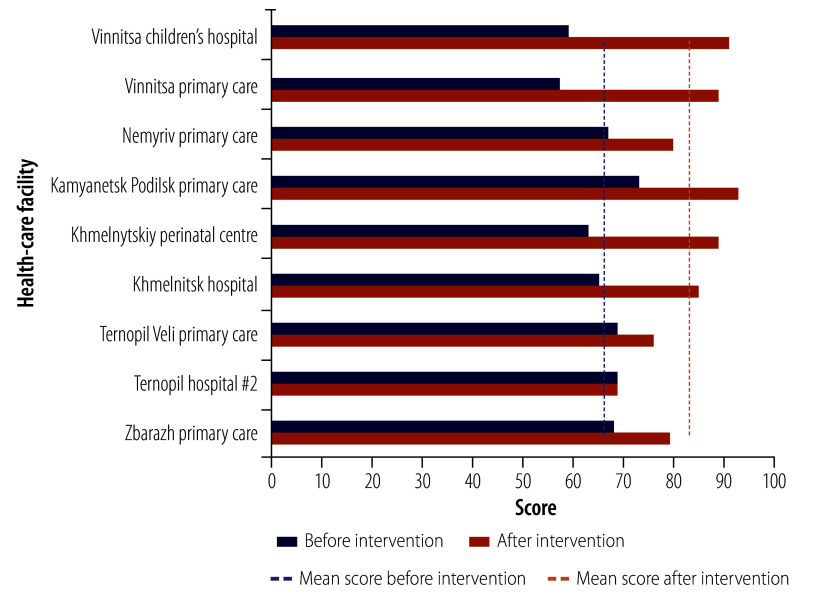
Water and Sanitation for Health Facility Improvement tool scores before and after intervention, Ukraine, March 2023 and July 2023

The baseline assessment identified that waste-handling practices were inadequate and did not align with new Ukrainian waste standards,^9^ including issues with segregation, irregular collection and treatment, and disinfection – rather than segregation and treatment – of all infectious waste. The assessment also identified issues with safely managing stored water and drinking-water availability. The average score of the assessment was 66 out of the maximum 100 (range: 57–73). In July 2023, six months after implementing the improvement plans in January 2023, the average score had increased by an average of 17 points to 83 (range: 69–93). All nine facilities improved their overall score. Improvements were greatest in health-care waste (30 points) and hand hygiene (31 points), and least in sanitation (7 points). Eight facilities had a total score above 75 (Fig. 1).

The assessment catalysed facilities to make positive changes, including installing water metres to monitor usage and encourage conservation; obtaining water quality data from suppliers; replacing broken taps; and replacing ineffective chlorine disinfection of waste with safe segregation and treatment. They also developed backup water supplies, such as drilling boreholes.

Improvements were made unequally across the facilities. Two hospitals and one primary care facility increased their score by 40% or more compared to baseline. By engaging facility and municipality leaders, these facilities mobilized public resources for additional improvements, for example to upgrade plumbing. Strong commitment of hospital leadership with high levels of technical knowledge and skills of multidisciplinary teams in the three health-care facilities were critical to improvements. Integration of water, sanitation and hygiene activities with infection prevention and control and antimicrobial resistance programmes at facility level also contributed to the progress. Less progress was observed in specialized, in-patient hospitals, where substantial funding is needed to improve water, sanitation and hygiene services for more advanced service needs. 

In October 2023, WHO, United States CDC and the health ministry hosted a national strategic meeting on water, sanitation, hygiene and health. The meeting included staff from three top-performing facilities as well as facilities from eastern Ukraine, and offered an opportunity to plan for longer-term investment needs and capacity support. 

## Lessons learnt

WASH FIT can be used to comprehensively identify gaps in water, sanitation, hygiene and waste services and track progress towards addressing these gaps. Strengthening water, sanitation and hygiene, and more broadly electricity services is an important part of emergency preparedness and response during war. Box 1 summarizes the main lessons learnt from this work.

Box 1 **Summary of main lessons learnt**

- Conducting thorough assessments with committed facility staff and multidisciplinary teams can reveal hidden issues and help build consensus on how to address critical gaps
- Small, more easily addressed improvements can be made with few resources, catalysing larger improvements and investment from municipalities and donors
- Building capacity and allocating resources for responsible government entities (e.g. regional CDC, Ukrainian Public Health Centre) to support implementation, surveillance and follow-up is critical to institutionalize the process

CDC: Centers for Disease Control and Prevention; UNICEF: United Nations Children’s Fund; WASH FIT: Water and Sanitation for Health Facility Improvement tool; WHO: World Health Organization.

The main challenges in implementation include budget constraints due to the war; staff turnaround, including in implementation teams; and multiple and unclear roles among government agencies in terms of monitoring and regulating water, sanitation and hygiene services in health-care facilities. WHO and United States CDC have recommended further external evaluations of water, sanitation and hygiene services in the initial nine pilot facilities to assess sustainability of changes, and any impact on infection prevention and control and antimicrobial resistance efforts.

In early 2024, implementation of WASH FIT with WHO and United States CDC support was expanded to nine facilities in eastern and southern Ukraine. WHO engagement with the government and partners has resulted in over 100 health-care facilities now implementing WASH FIT and making incremental improvements. Scale-up of WASH FIT, facilitated by the health ministry, Ukrainian Public Health Centre and regional CDCs, is coupled with reviewing and updating existing standards, more regular monitoring of services nationwide, including through the Health Resources and Services Availability Monitoring System tool which has been aligned with WASH FIT indicators. Efforts are also underway to build a national cadre of expert facilitators from the health ministry and regional CDCs, who will train health-care facility staff to use WASH FIT, conduct follow-up visits and link these activities more closely with ongoing efforts to improve infection prevention and control and reduce antimicrobial resistance.

The use of risk-based approaches, including WASH FIT, should be integrated into the ongoing humanitarian response and recovery plans worldwide, as well as longer-term reform strategies. Use of WASH FIT complies with new Ukrainian health-care waste standards. Implementing and meeting these new standards will require careful planning and must be guided by integrated strategies across health, infrastructure, environment, energy and economic sectors. To facilitate uptake, WASH FIT should be institutionalized in national legislations and be accompanied by systematic capacity-building and longer-term professional development. The adoption of risk-based approaches for water, sanitation and waste services are in line with current EU regulations and WHO health-based recommendations. In the future, closer monitoring of outcomes, including on quality and experience of care and on the growing problem of antimicrobial resistance will be important.
